# Trend in serological and molecular diagnostic methods for *Toxoplasma gondii* infection

**DOI:** 10.1186/s40001-024-02055-4

**Published:** 2024-10-28

**Authors:** Min-ju Kim, Soeun J. Park, Hyunwoo Park

**Affiliations:** 1 Health Park Co., Ltd, Seoul, 02447 Republic of Korea; 2 Epigenix Innovation, Destin, Florida 32541 USA; 3 Niceville High School, Niceville, Florida 32578 USA

**Keywords:** *Toxoplasma gondii*, Toxoplasmosis, Diagnosis, Specificity, Sensitivity

## Abstract

**Background:**

*Toxoplasma gondii*, an intracellular parasite, is a significant cause of zoonotic disease, with an estimated one-third of the world’s human population believed to be infected. *T. gondii* is transmitted to humans through the consumption of contaminated water, soil, vegetables, fruits, shellfish or undercooked meat, and can also be passed from human to human through vertical transmission, transplants and blood transfusion. While *T. gondii* infection typically manifests mild symptoms such as colds among immunocompetent individuals, it can prove lethal for those with weakened immune systems.

**Methods:**

To summarize the diagnostic methods for *Toxoplasma gondii* infection, we performed a literature search on PubMed from 1948 to 2023 using the keywords “*T. gondii* serological diagnosis” or “*T. gondii* molecular diagnosis”.

**Results:**

Rapid and accurate diagnosis of *T. gondii* infection is imperative. Although a diagnostic kit is currently commercially available, there are a number of disadvantages to the validation principles applied to each diagnostic kit. Consequently, multiple diagnostic methods are concurrently employed to offset these limitations. Serological methods for diagnosing *T. gondii* infection include the Dye Test (DT), Agglutination Test (AT), Modified Agglutination Test (MAT), Latex Agglutination Test (LAT), Enzyme-Linked Immunosorbent Assay (ELISA), and Western Blot. Meanwhile, molecular methods such as polymerase chain reaction (PCR), nested PCR, real-time PCR, loop-mediated isothermal amplification (LAMP), multiplex PCR, and PCR–restriction fragment length polymorphism (PCR–RFLP) are also utilized. Each of these methods possess its own set of advantages and disadvantages.

**Conclusions:**

By summarizing the advantages and disadvantages of different diagnostic techniques, it is hoped that the epidemiology, prevention, and control of toxoplasmosis will be improved in the future through the use of appropriate technologies.

## Introduction

*Toxoplasma gondii* is a zoonotic parasite that primarily infects warm-blooded animals, including humans, with reports indicating that one-third of the global human population is affected [1, 2]. Cats serve as the definitive host, and humans become infected through the consumption of uncooked meat from animals such as sheep, goat, or pigs that are infected with *T. gondii*, as well as through ingestion with soil or water contaminated with the parasite’s oocysts, often found in infected cat feces [3–6]. Transmission can also occur through vertical transmission, transplantation and blood transfusion involving an infected human donor [7]. *T. gondii* exhibits three primary parasite morphologies: oocyst, tachyzoite, and bradyzoite. In the intestines of its definitive host, the cat, *T. gondii* predominantly exists in the oocyst form [8]. However, in water, soil, or within other animals, it can be found as a tachyzoite [9] or as an encysted form in tissue [10]. The bradyzoite form is resistant to gastric juices and is considered the most critical form in the *T. gondii* life cycle [11].

While generally patients typically experience only mild symptoms such as cold-like symptoms [12], the elderly, children and immunocompromised individuals may develop severe symptoms such as neurotoxoplasmosis, ocular toxoplasmosis, and even death [13, 14]. Especially, the infected pregnant patients and newborns has symptoms abortion, stillbirth and miscarriage [3, 15]. Recent studies have also suggested a potential link between *T. gondii* infection and mental illness [16–18]. Therefore, it is crucial to swiftly and accurately diagnose *T. gondii* before these serious symptoms manifest in immunocompromised patients, particularly pregnant women [19], newborns [20], and HIV-infected individuals [21]. This is very helpful in determining the timing of drug treatment for each patient.

The lack of specificity in the clinical manifestations of toxoplasmosis underscores the imperative for accurate diagnosis employing *T. gondii*-specific diagnostic methods [22–24]. Furthermore, epidemiological investigations are expanding beyond human populations to include other mammalian hosts in the pursuit of preventive strategies. Historically, *T. gondii*-specific diagnostic assays have primarily been conducted in laboratory settings, encompassing parasite detection or detection of specific antibodies such as the Sabin–Feldman dye test (DT), indirect fluorescent antibody test (IFAT), agglutination test (AT), Western blot, and enzyme-linked immunosorbent assay (ELISA). Nevertheless, these diagnostic methodologies present several limitations, including reliance on live tachyzoites, detection of false-positives or cross-reactions, and the complex *T. gondii* cycle. To address some of these shortcomings, such as the timing of *T. gondii* infection, the genotype of the infected *T. gondii*, and the quantification of tachyzoites, diagnostic approaches such as polymerase chain reaction (PCR) have emerged, enabling the diagnosis of *T. gondii* infection through molecular techniques. Despite the commercialization of diagnostic kits based on these methods, challenges such as cross-reactivity leading to false-positive results remain [25–27]. Despite the inherent complexities in the life cycle and strains of *T. gondii*, ongoing research endeavors into diagnostic methodologies aim to surmount these obstacles and delineate future avenues for improved diagnosis of *T. gondii* infection. This review seeks to comprehensively elucidate each of the *T. gondii* diagnostic modalities.

## Dye test (DT)

The Sabin–Feldman test or the Dye Test (DT) is a traditional diagnostic method developed over 70 years ago and primarily conducted in laboratory settings. Despite the time it has been in use, this is one of the most popular diagnostics still in use [28, 29]. It has been used in epidemiological studies in sheep and cats [30, 31]. In this method, live tachyzoites are incubated with serum from a suspect sample and then stained with methylene blue. The basic principle is that *T. gondii*-specific neutralizing antibodies and complement factors in the serum damage the external structure of live tachyzoites, preventing them from staining [28, 32]. Determination of antibody titer involves calculation of the ratio of live to dead tachyzoites or serum sample titer as the highest dilution at which the sample tests positive [29, 33]. However, due to its reliance on live tachyzoites, this method is limited to laboratory settings and requires skilled operators. It is also associated with a risk of false-negative and false-positive results [33].

## Agglutination test (AT)

Unlike the DT, which uses live tachyzoites, the agglutination test is performed on U-shaped microtitre plates using tachyzoites inactivated with chloroform or formaldehyde together with antibodies from suspect samples. Positive (thin mat formation) or negative (compact pellet pattern) results are determined from the aggregation pattern observed on the bottom of the U-shaped plate [34]. Various modifications of agglutination tests such as direct agglutination test (DAT), modified agglutination test (MAT), indirect haemagglutination test (IHAT) and latex agglutination test (LAT) have been developed for the diagnosis of toxoplasmosis. DAT, the first agglutination test developed in 1959, has the advantage of ease of testing, as it does not require any additional specimens or equipment compared to DT for qualitative results, using inactivated tachyzoites [34, 35]. However, it suffers from low specificity and sensitivity due to non-specific IgM antibody binding [36, 37]. To overcome these limitations, modifications have been made to DAT by altering the tachyzoite or adding experimental substances. MAT incorporates 0.2 M 2-mercaptoethanol to prevent destruction by non-specific IgM antibodies [38], although it can still give false-positive results [39, 40]. IHAT uses *T. gondii* specific IgM antibodies for the serodiagnosis of acute toxoplasmosis [41], using 2-mercaptoethanol treated serum samples from infected patients, inactivated tachyzoites and human red blood cells [42]. IHAT has a lower relative sensitivity than MAT, despite similar concordance rates [43]. Conversely, LAT increases the sensitivity and specificity of conventional AT by covalently binding tachyzoites to latex particles [44]. Sensitivity and specificity were found to be high when latex particles were diluted in AMP buffer and 0.2 M 2-amino-2-methyl-1-propanol in a 1:5 ratio [45], although the problem of false positives remains [46].

## Western blot

Western blot (immunoblot) is a technique used to detect specific molecular weight bands of *T. gondii* antigenic proteins by loading *Toxoplasma gondii* tachyzoite lysate antigen or recombinant protein antigens onto a polyacrylamide gel and then incubating with antibodies from a suspect sample [47]. This method is capable of detecting both IgG and IgM antibodies and is used to diagnose not only congenital toxoplasmosis but also post-natal infection in samples from pregnant women, mothers and newborns [47, 48]. Studies have investigated antibody detection using recombinant GRA2 protein instead of *T. gondii* tachyzoite lysate antigen [49]. In addition, sensitivity and specificity have been evaluated using the recombinant protein antigen MAG1 expressed in *E. coli* to increase the binding affinity of IgG and IgM antibodies [50]. Western blot diagnostic methods have also been evaluated using similarly prepared recombinant protein antigens such as SAG1 and GRA7 [51]. While commercial diagnostic kits based on western blot methods are available, they have demonstrated relatively low sensitivity compared to other diagnostic techniques [52, 53].

## Enzyme-linked immunosorbent assay (ELISA)

ELISA, a widely used diagnostic method among serological techniques, has high sensitivity and specificity and is widely used in laboratories. Various types of ELISA using *T. gondii* tachyzoite lysate antigen, excretion/secretion antigen, chimera/recombinant protein antigen or *T. gondii*-specific antibodies have facilitated the detection of antibodies or antigen in samples such as serum and plasma (Table 1). Diagnosis is primarily based on the detection of *T. gondii* specific IgG or IgM in laboratory samples by this method [54]. The timing of IgG and IgM antibody production after infection with *T. gondii* varies, leading to the classification of acute and chronic infections based on IgG and IgM measurements. IgM is detected in acute infections, but because IgM can persist for up to a year, the diagnosis of acute and chronic infections is made by measuring IgG levels simultaneously rather than relying on IgM detection alone [55–57]. With the improvement in diagnostic sensitivity, the IgM assay is now followed by an IgG antibody avidity test to calculate a percentage that provides a general estimate of the time of infection [56]. Commercial ELISA diagnostic kits are useful not only for clinical samples [58, 59] but also for preventive measures in animal such as sheep and pigs [60–64].

**Table 1:** Type of serological methods for toxoplasmosis diagnosis

Technique(s)	Antigens or antibodies	Advantage(s)	Disadvantage(s)	References
Dye test (DT)	Tachyzoites		Live tachyzoites are needed, experienced experimenters can only perform, potential for false negative and false positive results	[28–31, 33, 167]
Agglutination test (AT)	Tachyzoites	Killed tachyzoites are needed, not require any additional experiments	Lower sensitivity and specificity than ELISA, potential for false positive results	[34]
Modified agglutination test (MAT)	Tachyzoites added 0.2 M 2-mercaptoethanol	Relatively highly specificity	Potential for false positive results	[38–40]
Latex agglutination test (LAT)	Tachyzoite covalently bound to latex particles	Relatively highly sensitive and specificity	Potential for false positive results	[44–46]
Western blot	Tachyzoites lysate antigen, recombinant and chimeric proteins	Detection of IgG, IgM, IgA and IgE	Lower sensitivity and specificity than ELISA, depending on the serum being diagnosed	[47, 48]
Indirect ELISA (iELISA)	Tachyzoite lysate antigen, recombinant and chimeric proteins	Detection of IgG and IgM, a large number of samples can be performed simultaneously	Potential for false positive results, not suitable for detection of genotype	[48, 51, 54, 58, 60, 62, 65–70, 73–88, 92–97, 168]
Nano-ELISA	SAG pAb-AuNP, E/S antigen-AuNP	Highly sensitive and specificity		[102, 103]
Sandwich ELISA	Recombinant protein, capture antibodies	Detection of IgG and IgM, a large number of samples can be performed simultaneously	Potential false positive results, not suitable for detection of genotype	[54, 98]
Indirect fluorescent antibody test (IFAT)	Tachyzoites, anti-*T. gondii* antibodies conjugated fluorescent	Relatively highly sensitive	Expensive experiment and experience are needed, potential for false negative and positive results	[104, 105]
Immunochromatographic test (ICT)	Recombinant protein, antibodies	Rapid and more convenient to use	Potential for false negative results	[107–110]
Immunosorbent agglutination assay (ISAGA)	Anti-human IgM antibodies	Highly sensitive and specificity, convenient to use	Potential for false positive results on patient samples for diseases such as HIV	[111]

Indirect ELISA involves coating the bottom of an immune plate with *T. gondii* antigens and incubating it with serum antibodies. These ELISA assays predominantly use *T. gondii* tachyzoite lysate antigen (TLA) for the detection of IgG or IgM antibodies and show substantial agreement with MAT, IHA or IFAT [60, 62, 65, 66]. However, similar to the PCR diagnostic approach, ELISA using TLA has limitations, including potential contamination by impure substances, as well as false negatives and false positives resulting from the purification process of parasites cultured from living mice and cells [58, 67–70], and cross-reactivity with antigens/proteins between *T. gondii* and other closely related parasites [25–27]. In addition, Result of false positives were caused. Despite these drawbacks, it remains the most widely used diagnostic method due to its ability to test a large number of samples at the same time and to detect both IgG and IgM antibodies.

In recent years, there has been research into ELISA assays using recombinant antigens, which have demonstrated high sensitivity and specificity in the detection of *T. gondii*-specific IgG or IgM antibodies [27, 71, 72]. In addition to improved sensitivity and specificity, ELISAs using recombinant antigens have the advantage of differentiating between acute and chronic toxoplasmosis [69, 73]. The recombinant antigens studied include surface antigens involved in host cell invasion, such as SAG1 [51, 74–76], SAG2 [77–79] and SAG3 [48], as well as dense granule antigens (GRAs), secretory proteins expressed during the tachyzoite, bradyzoite and sporozoite stages of *T. gondii* that are being investigated for their role in the generation and maintenance of the parasitophorous vacuole (PV) within cells for parasite persistence after infection, namely GRA1 [80–82], GRA4 [83] and GRA7 [84–86]. The antigens also include proteins such as the rhoptry antigen ROP2 [78] and apical membrane antigen 1 AMA1 [87, 88], which, like the SAGs, play a crucial role in intracellular invasion. In addition, modified proteins have been predominantly used as antigens, incorporating T or B cell epitopes of single antigens [89–91], chimeric protein consisting of multiple antigens [92–95], or influenza M1 virus-like particles containing *T. gondii* antigens [96, 97].

Sandwich ELISA detects antibody–antigen interactions by immobilising antibodies on the bottom of the plate and allowing them to react with antigens. In diagnostic procedures, the capture antibody is applied to the bottom of the plate and incubated with antibodies containing *T. gondii* antigen. This method is primarily used for antigen detection, although some have been developed for antibody detection, and is highly sensitive and specific for the detection of IgM antibodies than the immunofluorescence assay (IFA) and allows the diagnosis of congenital toxoplasmosis [54]. In addition, the sandwich ELISA provides a simple method for the detection of circulating antigens such as MIC10 and GRA1, which are important indicators for the diagnosis of acute infections and are difficult to detect by indirect ELISA [98–101].

Recent studies have investigated ELISAs using nano-gold synthesized with antigens or antibodies coated on plates to overcome the limitations of conventional ELISAs that has relatively low sensitivity and specificity. In one study, they developed a nanogold ELISA by synthesizing SAG1 pAb with AuNPs and compared its performance with that of conventional ELISA. The nano-gold ELISA showed a sensitivity of 89.2%, higher than the 83.8% observed with the conventional ELISA. In addition, the specificity and diagnostic accuracy were 94% and 91.95% respectively, also exceeding the conventional ELISA values of 88% and 86.2% [102]. In another study, a nano-ELISA was developed by synthesizing E/S antigen with AuNPs. This nano-ELISA achieved a sensitivity and specificity of 93.33%, significantly higher than the 80% sensitivity and 86.66% specificity of the conventional ELISA [103]. The improved performance is attributed to the increased surface area provided by the nanoparticles, which facilitates a greater number of antigen–antibody interactions.

## Other serological methods

In addition to the agglutination test (AT) and enzyme-linked immunosorbent assay (ELISA) mentioned above, other methods include the indirect fluorescent antibody test (IFAT), the immunochromatographic test (ICT) and the immunosorbent agglutination assay (ISAGA) (Table 1). In IFAT, serum from a sample is incubated with inactivated *T. gondii* tachyzoites, bradyzoites or oocysts, antibodies conjugated fluorescent are added and observed under a fluorescence microscope [104–106]. However, as this diagnostic method relies on visual observation, there may be operator variability. ICT, on the other hand, is a rapid diagnostic technique that uses antigens or antibodies on a cellulose membrane. Studies have used GRA7 [107, 108] and SAG3 [109] as antigens, with ICT having the advantage of being faster and more convenient than ELISA [110]. ISAGA is a method specifically for the detection of IgM antibodies. Anti-human IgM antibodies are fixed to the bottom of the plate, the serum sample is incubated and agglutination is confirmed by incubation with *T. gondii* tachyzoites, with the assay being similar to MAT [111].

## Polymerase chain reaction (PCR)

In contrast to serological diagnosis, PCR is a molecular diagnostic method that involves the amplification of genes specific to *T. gondii*. PCR can detect infections at very low concentrations and can quantify *T. gondii* tachyzoites in tissue samples that cannot be measured by serological analysis (Table 2). Serologic diagnosis can confirm infection in pregnant women and assess the risk of congenital toxoplasmosis. However, direct diagnosis of parasitic infections in infants is challenging due to the high risk of sample collection [55]. However, molecular techniques such as PCR allow testing not only of serum but also of samples from which DNA and mRNA can be extracted, including amniotic fluid, blood, brain tissue, eyes and urine, thereby facilitating confirmation of parasitic infection in infants [22, 112, 113].

**Table 2:** Type of molecular methods for toxoplasmosis diagnosis

Technique(s)	Target genes	Advantage(s)	Disadvantage(s)	References
Conventional PCR	B1, p30, 18S rDNA, rep529, SAG2, GRA2, ROP8, TGR1E	Suitable detection of genotype, high resolution in typing	Lower sensitivity than real-time PCR, potential for false negative results, isolated parasite needed	[112, 115, 117, 124, 129, 135, 136, 139, 157, 158]
Nested-PCR	B1, p30, 18S rDNA, ITS-1, SAG2, GRA7	Suitable detection of genotype, and isolation of parasites may not be needed	Lower sensitivity than real-time PCR, potential for false negative results	[112, 116, 118, 127, 130, 133, 134, 138–141]
Multiplex PCR	B1, SAG2, ITS-1	Suitable for epidemiology studies, high resolution in typing	Difficult to setup	[148]
Real-time PCR	B1, p30, 18S rDNA, rep529, SAG2, GRA2	Highly sensitive, rapid, isolation of parasites not needed	Expensive equipment is needed	[126, 132, 139, 142, 158]
LAMP	B1, p30, rep529, SAG2, GRA2, ITS-1	Highly sensitive, specific, rapid	Potential for false positive results, difficult primer designing	[131, 138, 146, 147]
PCR–RFLP	p30, SAG1, 2,3, BTUB, GRA6, C22-8, C29-2, L358, PK1, APICO	Suitable detection of genotype	Lower sensitivity than real-time PCR	[149, 150]
HRM	B1, ROP8	Rapid and highly sensitive, suitable detection of genotype	Difficult to set the temperature	[135]
Digital droplet PCR	rep529	Highly sensitive, more accurate quantification	Further research needed	[165, 166]

In the early stages of PCR diagnostics, the B1 gene was primarily used in the laboratory [114–118]. Other target genes such as p30, ribosomal DNA, rep529, GRA7 and ROP8 genes have been incorporated to increase sensitivity and specificity. The B1 gene, traditionally used in PCR diagnostics, is one of the most conserved genes in the *T. gondii* genome, making it an ideal candidate for PCR diagnostics due to its 35-fold repetition in the *T. gondii* genome [114, 117, 119]. In addition, the primer for the B1 gene exhibits specificity by selectively avoiding amplification of fungal, bacterial or human lymphocyte DNA [116]. However, recent studies have reported low specificity of the B1 gene [120, 121]. Currently, the B1 gene is used to diagnose *T. gondii* infection in immunocompromised patients such as pregnant women or those with AIDS [122–126]. Although the p30 gene offers greater sensitivity than B1 [127], research indicates that it has lower specificity compared to the B1 gene, as it may overlap with DNA from *Nocardia* and *Mycobacterium tuberculosis* [116, 128]. On the other hand, the rep529 gene, a fragment repeated 200–300 times in the *T. gondii* genome, has the ability to detect 60 strains among *T. gondii* strains [129]. The sensitivity of the rep529 gene exceeds that of the B1 gene [129–131], and its high sensitivity and specificity allow the detection of positive samples missed by currently commercialized PCR-based diagnostic kits [132]. Consequently, the B1 gene and the rep529 gene are commonly targeted because of these advantages, with ongoing PCR studies focusing on genes such as SAG2 [133], GRA7 [134], ROP8 [135, 136], and ITS-1 [137, 138] to further improve not only sensitivity but also specificity.

To further increase the sensitivity of conventional PCR, various target genes have been developed, as discussed above, along with simultaneous advances in nested-PCR [112, 116, 118, 127, 130, 133, 134, 138–141], real-time PCR [129, 142–144], real-time PCR–High-Resolution Melting Mechanism (PCR–HRM) [85, 135, 145], loop-mediated isothermal amplification (LAMP) [131, 138, 146, 147], multiplex PCR [148], and PCR–restriction fragment length polymorphism (PCR–RFLP) [149, 150]. PCR–HRM is a method for analyzing the DNA characteristics of each gene that is very simple and cost effective [151, 152]. This approach has the advantage of differentiating the genotype of *T. gondii* infection. However, it has been found that sensitivity varies depending on the target gene [135]. PCR–LAMP is an amplification method with high sensitivity and specificity for distinguishing single nucleotide differences [153]. In particular, it has been reported to be more sensitive than nested PCR when detecting DNA from blood samples [154, 155]. PCR–RFLP also has the advantage of genotyping *T. gondii*, which can be used to confirm the geographical patters and so the epidemiological dynamics of *T. gondii* genotypes [156].

The challenge of PCR-based diagnosis lies in the variability in sensitivity, specificity and results observed between different laboratories, each of which uses its own protocol for extracting DNA or mRNA, even when using the same primer [112, 139, 140, 157, 158]. Efforts to address this issue have included proposing standardization methods with a reference protocol, but complete standardization remains an ongoing goal [159]. In addition, the classification of *T. gondii* strains identified to date (over 950 genotypes) as type 1, 2, 3 or atypical strains that do not fit the classical classification cannot be conclusively determined using single gene assays [53, 160]. Therefore, assays using a combination of genes are required to accurately determine the genotype of an infected *T. gondii* [160–162].

Digital Droplet PCR (ddPCR) has recently been developed to enhance the utility of PCR. This technique directly quantifies parasitic infections by maximizing the amplification of single DNA templates, thereby increasing sensitivity and specificity for detecting low-level parasite load. It produces a linear digital signal DNA product, enabling the detection of mutations [163]. These capabilities allow for the detection of low-level parasite load and differentiation between related parasite species, making ddPCR a method of significant interest in clinical diagnosis and screening [164, 165]. In one study, ddPCR demonstrated high sensitivity and specificity compared to real-time PCR, achieving 100% sensitivity and specificity in 80 mussel DNA samples [166]. The primary advantage of ddPCR is its accurate quantification, which addresses the quantification inaccuracies often encountered with qPCR [165].

## Conclusion

The purpose of this review is to provide an overview of the different diagnostic methods for toxoplasmosis infection that are currently in development, as well as their advantages and disadvantages. Early detection of toxoplasmosis, especially in immunocompromised individuals such as pregnant women or people with HIV, is critical to prevent progression to severe disease through rapid and accurate diagnosis. In addition, because *T. gondii* is a zoonotic parasite, a key goal is to prevent human infection by diagnosing and detecting it in all matrices in which it may be present, including humans, mammals, birds, water, soil, vegetables, fruits and shellfish. A variety of diagnostic methods are currently being used to achieve this, with serological and molecular diagnostics being favored over techniques that directly detect the parasite. Understanding the advantages and disadvantages of the different diagnostic techniques presented in this review can help not only in preventing infections, but also in diagnosing future infections and choosing the most effective treatment.

## Data Availability

No datasets were generated or analysed during the current study.
